# Beyond Treatment: Electrochemical Phenol Oxidation in Methanol under Different Cell Configurations and Its Implications for Valorization

**DOI:** 10.1021/acsomega.6c01902

**Published:** 2026-04-30

**Authors:** William Santacruz, Cristina Navas-Higuero, Cristina Saez, Artur de Jesus Motheo, Manuel Andrés Rodrigo

**Affiliations:** † São Carlos Institute of Chemistry, University of São Paulo, São Carlos, São Paulo 13566-590, Brazil; ‡ Department of Chemical Engineering, 16733 Universidad de Castilla-La Mancha, Ciudad Real 13071, Spain

## Abstract

This study explores electrochemical phenol oxidation in methanolic and aqueous media as a strategy to shift adsorption-based treatments from pollutant removal toward chemical valorization. Using 3D-printed single- and divided-cell reactors with alkaline electrolytes to enhance conductivity, methanol clearly outperformed water: phenol conversions exceeded 50% after 3 h (vs <15% in water), with higher Faradaic efficiencies (>21%) and energy efficiencies up to 3-fold greater, especially in anion exchange membrane (AEM) systems. Speciation analysis revealed that methanol favors diverse, industrially relevant intermediates, including carboxylates (oxalate, malonate, succinate, tartrate, maleate) and methoxylated aromatics, whereas aqueous media predominantly formed benzoquinone and tetrahydroxybenzene, consistent with rapid hydroxyl-radical-driven mineralization. The strong influence of membrane type on phenol transport (AEM > PEM) and the distinct degradation pathways observed (hydroxylation/cleavage in water vs methoxylation/fragmentation in methanol) highlight the critical role of solvent–membrane coupling. Overall, the results demonstrate that electrochemical processing in methanol promotes the formation of identifiable value-added products, indicating potential implications for future resource-oriented wastewater treatment strategies.

## Graphical abstract

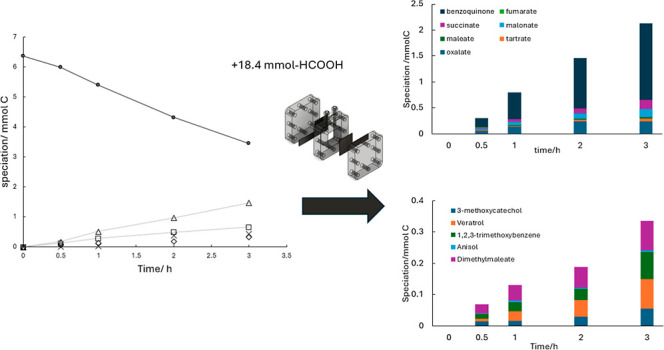

## 1 Introduction

Recently, increasing the concentration of organic contaminants in aqueous waste streams prior to electrochemical treatment has been proposed as a strategy to enhance the efficiency of electrochemical wastewater treatment processes, particularly those characterized by low organic load and low ionic strength.^1–4^ This approach is based on the fact that, in most cases, process efficiency is strongly dependent on contaminant concentration, as their degradation generally follows first-order kinetics. Consequently, applying the same electrical charge yields the same percentage of contaminant removal regardless of the initial concentration, making preconcentration techniques highly beneficial for improving overall process performance allowing them to reach extremely higher efficiencies. In fact, this improved behavior has been demonstrated for ionic contaminants using electrodialysis as a concentration technique^5,6^ and for colloidal contaminants using coagulation and/or electrocoagulation.^7^

However, the case that may attract the greatest interest, because it is not strongly associated with specific characteristics of organic contaminants, is linked to the integrated process known as methanol-based electrochemically assisted adsorption,^8,9^ which belongs a range of processes that combine reaction and separation technologies into a unified operation.^10–14^ The most effective way to operate this process consists of sequential adsorption onto granular activated carbon (GAC) followed by regeneration of the adsorbent with methanol, a solvent that, in a final stage, is electrochemically regenerated to close the cycle and enable methanol reuse.^15^ This last stage, electrochemical regeneration, is the most critical for ensuring sustainability. Although alcohol is less polar than water, it can still serve as a supporting electrolyte in electrochemical degradation processes efficiently. Combined with the more favorable equilibrium for desorption of organic contaminants from activated carbon compared to water, this enables the concentration of contaminants in methanolic solutions at levels several folds higher than those in the original wastewater, significantly improving overall treatment efficiency.^8,16^

Moreover, the use of electrochemically assisted adsorption with GAC and methanol has proven to be not only effective for treating wastewater with low contaminant concentrations but also for gas treatment, as volatile organic compounds (VOCs) present in gases are efficiently retained on activated carbon and subsequently recovered with methanol, allowing regeneration of the solvent after contaminant removal.^9,16–18^ Therefore, this process can have a highly relevant impact on environmental remediation, as demonstrated in various studies involving contaminants such as perchloroethylene, among others.^19^

In this context, one of the most important observations made when using this technology, besides efficient contaminant destruction, is the methoxylation of contaminant molecules, primarily resulting from the transformation of methanol into methoxy radicals.^20,21^ It is important to note that, unlike oxidation in aqueous media, where hydroxyl radicals largely drive process efficiency, oxidation in methanolic media is not influenced by these radicals but occurs either directly on the electrode surface or mediated by oxidants generated from ions present in the methanolic solution. Far from being a drawback, this process may be highly interesting, as methoxylation frequently produces molecules with significant economic value, forming the basis of numerous industries such as fragrance production and other specialty chemicals^22–26^

This concept can be integrated into the recently proposed electro refinery process,^27,28^ which aims to achieve a paradigm shift in environmental treatment of contaminated streams by prioritizing the transformation of organic contaminants into valuable molecules rather than their conventional mineralization into CO_2_.^29^ In the primary concept of organic electro refinery, this shift is associated with the generation of carboxylic acids and their concentration using electrodialysis.^30–32^ However, in methanolic media, the potential goes much further, involving the formation of other species with inherent added value (such as toluene derivatives) and the possible formation of esters through interaction of generated carboxylates with methanol, which could have a significant impact on the novel circular economy concepts.^33^

All these considerations highlight the need for studies that allow a deeper understanding of the nature of electrochemical contaminant treatment processes in methanolic media in order to advance its development and application, now that the technological concept has been proven viable. As is always the case when working with low technology readiness levels (TRL technologies), it is necessary to start with simple model molecules that facilitate the extraction of clear conclusions.^34^ In this context, phenol has traditionally been used as a reference model for anthropogenic environmental contamination due to its aromaticity and non-negligible water solubility, with oxidative mechanisms and intermediates well-known for decades under various advanced oxidation processes (AOPs), including electrochemical processes.^35,36^

In this context, the present work aims to advance the understanding of electrochemical contaminant treatment in methanolic media by the electrochemical oxidation of phenol in methanolic and aqueous media, assessing the influence of solvent and membrane compartmentalization, with a particular focus on promoting conversion toward recoverable highly added value (HAV) products rather than complete mineralization to CO_2_, and on identifying the nature of the intermediates formed, thereby evaluating the potential of methanolic systems within an electrorefinery-oriented remediation framework. To avoid interferences in the interpretation of the results that could arise from the presence of anions such as chlorides or sulfates, whose oxidation can generate reactive chlorine species or persulfates with a substantial impact on process efficiencies, sodium hydroxide was used as the sole ionic component to increase the electrical conductivity of both supporting electrolytes. Under the operating conditions employed, the sodium cation remains inert; therefore, this base only promotes the formation of oxygen-based oxidants, particularly hydroxyl radicals and their derived species. This applies both to methanolic media, where the interaction between hydroxyl radicals and methanol is well established, and to aqueous media, where the role of hydroxyl radicals in the formation of hydrogen peroxide and ozone has been extensively described.

## 2 Materials and Methods

### 2.1 Experimental Setup

Figure 1 shows the experimental setup used for the tests presented in this work, both in its operation with a divided cell and with a single-compartment cell. It was printed using an acrylic-based resin (Clear Resin V4, FormLabs) in a Form 3 machine procured from Formlabs. Under the experimental conditions employed in this study, the material showed adequate stability for reactor operation.

**Figure 1:**
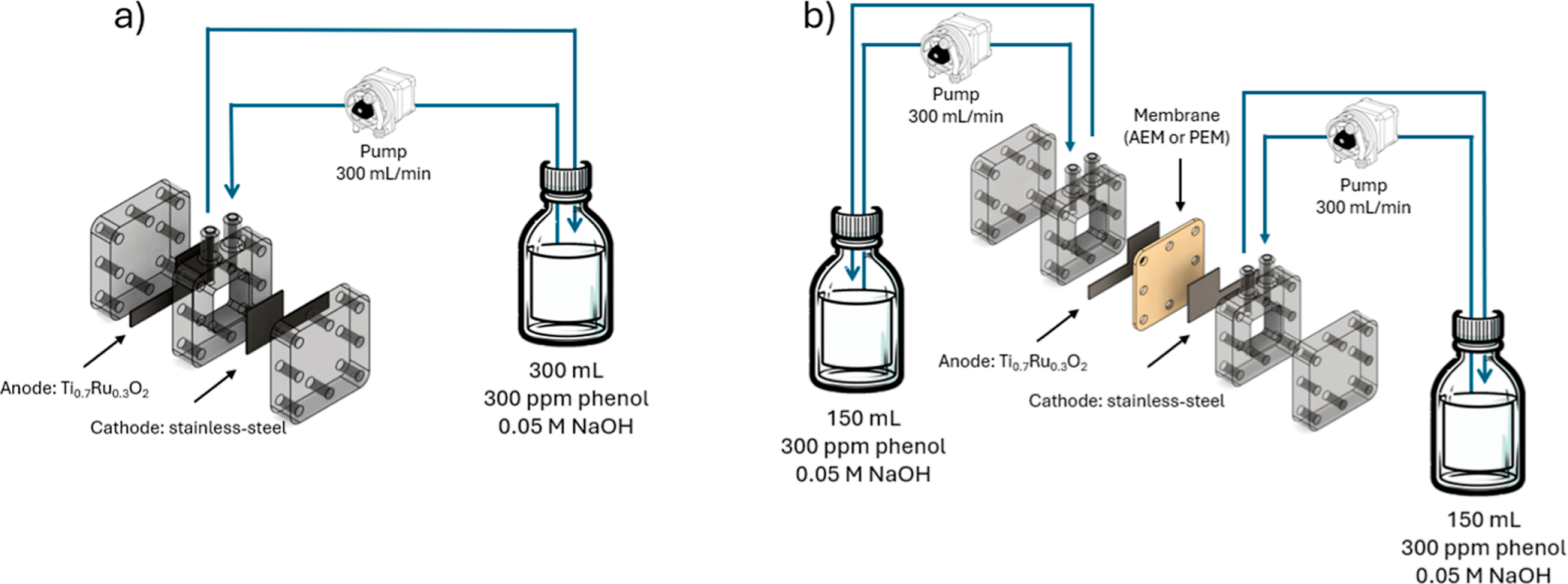
Experimental setup. (a) single-compartment cell. (b) bicompartment cell.

The cell was equipped with a mixed metal oxide anode (Ti_0.7_Ru_0.3_O_2_) and a stainless-steel cathode, with both electrodes measuring 2.0 × 2.0 cm^2^. The 3D printed cell design corresponds to a flow cell, with each compartment connected to a reservoir containing the liquid to be processed. This liquid initially has the same composition, whether processed as a single stream (undivided cell) or fed into the divided cell, and in both cases, the process is operated in batch mode with tests lasting 3 h. This means that the specific electrical charge applied at a given current density is the same over time in all experiments, allowing for direct comparison of results. The treated volume is always 300 mL of an aqueous or methanolic phenol solution at 0.3 g L^–1^, with a 50 mM of sodium hydroxide. A proton exchange membrane (PEM) PFSA D50-R ePTFE (from fuel cell store) and an anion exchange membrane (AEM)—AMH PES (from Ralex) were used to separate the electrodes in the bicompartment cell.

## 3 Results and Discussions

In previous experiments conducted as part of this work, the formation of formate was observed during the oxidation of methanolic solutions containing 0.05 M of sodium hydroxide. This type of process was also reported in literature.^37^ For this reason, a preliminary characterization study of this process was carried out to determine its extent and establish baseline conditions for the electrochemical oxidation of phenol in methanolic solutions.

### 3.1 Formate Formation and the Effect of Cell Configuration

The results of three of the electrolysis included in that study are shown in Figure 2, where the formation of significant amounts of formate are observed during the electrochemical processing of 150 mL of a methanol/NaOH (50 mM) solution under current density of 50 mA·cm^–2^ reaching concentrations of 590.3 mg·L^–1^ in a single cell, 634.8 mg·L^–1^ in the anodic compartment of an electrochemical cell equipped with an AEM and 535.4 mg·L^–1^ in the anodic compartment of an electrochemical cell equipped with an PEM respectively, after 3 h of electrolysis. In the single compartment electrolyzer, it is observed an almost linear trend over time, while in the AEM-electrolyzer the rate (which was initially higher) decreases over time and in the PEM-electrolyzer it showed the opposite behavior and the formation of formate speed up after the first moments in which no formate was produced.

**Figure 2:**
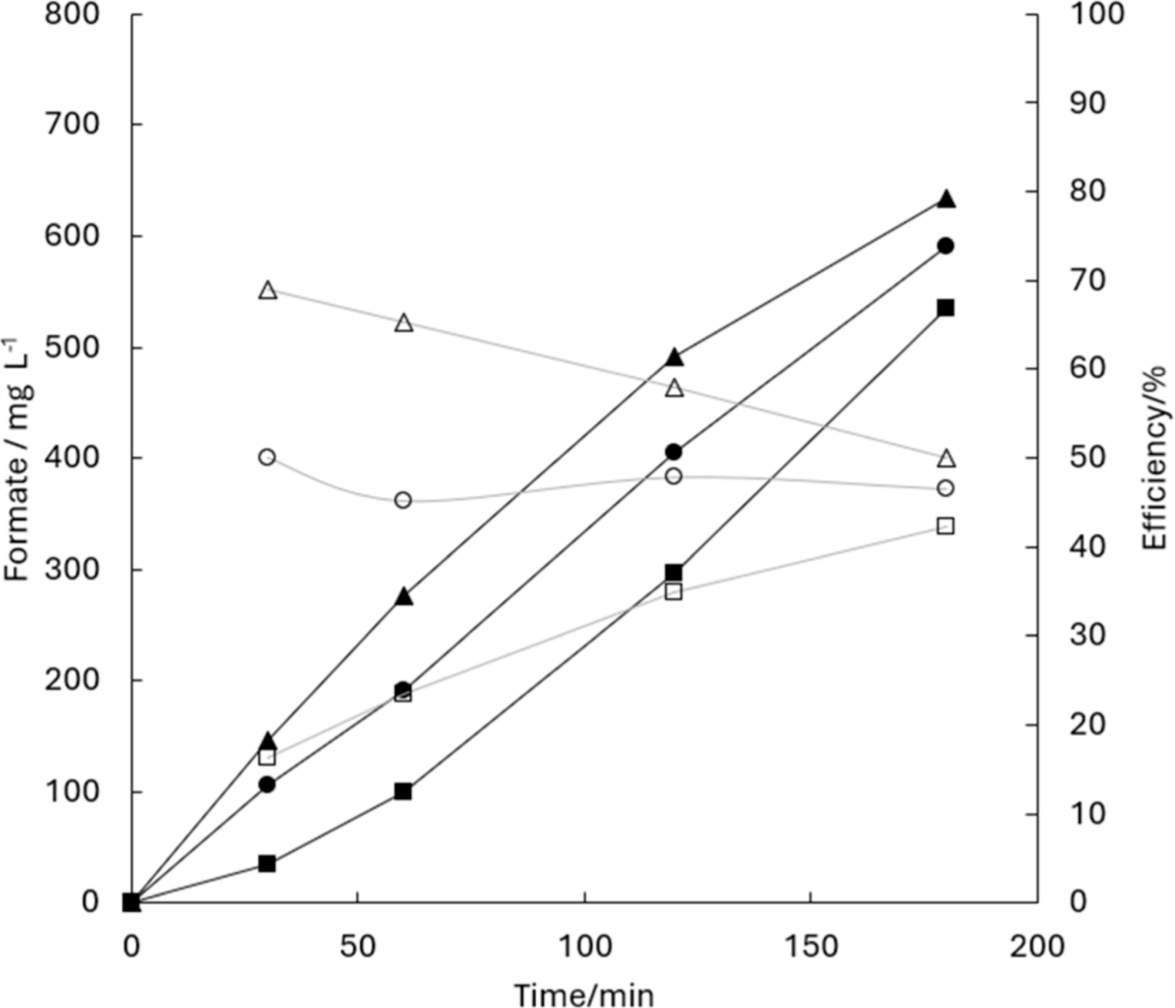
Evolution of formate concentration (filled symbols) and current efficiency (open symbols) in different cell configurations: single-compartment cell (●, ○), divided cell with an AEM (▲, △), and divided cell with a PEM (■, □) (50 mM NaOH; *j* = 50 mA cm^–2^).

Figure 2 also shows the evolution of current efficiency for formate formation, calculated according to the stoichiometry of methanol oxidation to formic acid given in eq 1. The profiles indicate that formate concentration increases with electrolysis time in all cell configurations in methanolic medium, following the order AEM > single-compartment cell > PEM. Regarding current efficiency, distinct trends are observed: it decreases in the AEM system, remains nearly constant in the single-compartment cell, and increases almost linearly in the PEM system. These results indicate that, although formate continues to accumulate in all cases, the fraction of the applied current effectively directed to its formation changes depending on the cell configuration, reflecting the competition with parallel reactions such as oxygen evolution or complete oxidation to CO_2_.

1 $${CH}_{3}OH+H_{2}O\to HCOOH+4H^{+}+4e^{-}$$

2 $$H_{2}O\to 0.5O_{2}+2H^{+}+2e^{-}$$

The results must be explained based on mechanisms of direct oxidation (methanol oxidation reaction, MOR) and/or hydroxyl-radical oxidation, since the hydroxide ions added through sodium hydroxide can be easily oxidized in the system to this type of radicals. Although in the gas phase methanol leads to formaldehyde through the formation of the methoxy radical, in the liquid phase the reaction continues until formate is produced.^38^ In contrast, direct oxidation is usually more closely associated with the formation of carbon dioxide and depends more deeply on the adsorption of methanol onto the anode.^39^

In this context, the same solution was introduced into both the cathodic and anodic compartments of the AEM electrolyzer. This configuration allows a larger amount of hydroxide ions to migrate toward the anode by electromigration, thereby increasing the reaction rate, as this radical precursor is the limiting species in the radical-mediated oxidation pathway. This explains the faster reaction rate observed compared with the single-compartment electrolyzer.

Conversely, the proton-exchange membrane does not permit the hydroxide ions present in the cathodic compartment to cross into the anodic side; at most, they may be consumed at the cathode through hydrogen evolution. This can drive ion migration in the opposite direction and, consequently, lead to the initial phase of low reactivity observed in this system.

Despite starting from very different initial conditions, the yields obtained after 3 h of electrolysis in all three configurations converge to approximately 46%. This suggests that the direct oxidation pathway leading to CO_2_ also plays a significant role in the overall process. However, because the formation of formate is not the focus of this work, and has already been extensively studied, it was not considered necessary to explore this pathway further. In addition, since this carboxylate formation does not depend on the electrochemical processing of phenol but rather on the solvent, in the following sections the evolution of formate concentration will be treated separately, as it is equivalent in methanolic media to what water oxidation represents in aqueous media for oxygen and proton formation (shown in Equation 2). Both reactions are considered competitive processes in this work with respect to phenol degradation, which is evaluated as the model contaminant.^39–41^

### 3.2 Phenol Degradation in Single-Compartment Electrochemical Cell

Figure 3 compares the electrolysis results obtained in methanolic medium (Figure 3a) and aqueous medium (Figure 3b) for phenolic solutions treated in single-compartment electrochemical reactors. For clarity, all detected species have been grouped into four categories (in addition to the original phenol): methoxylated species, nonmethoxylated aliphatic species (carboxylates), nonmethoxylated aromatic species, and carbon dioxide. Examination of the experimental data reveals the unexpected result that phenol transformation is more extensive in the methanolic medium than in the aqueous medium. In methanol, the formation of nonmethoxylated aromatic intermediates (such as benzoquinone) and nonmethoxylated aliphatic intermediates (carboxylates) predominates, with the latter produced in proportions comparable to methoxylated intermediates and CO_2_.^42,43^ In contrast, in the aqueous medium, mineralization is the dominant pathway,^36,44–46^ and intermediate species are detected at much lower concentrations,^47^ practically negligible making difficult, but not impossible, their valorization.^33^

**Figure 3:**
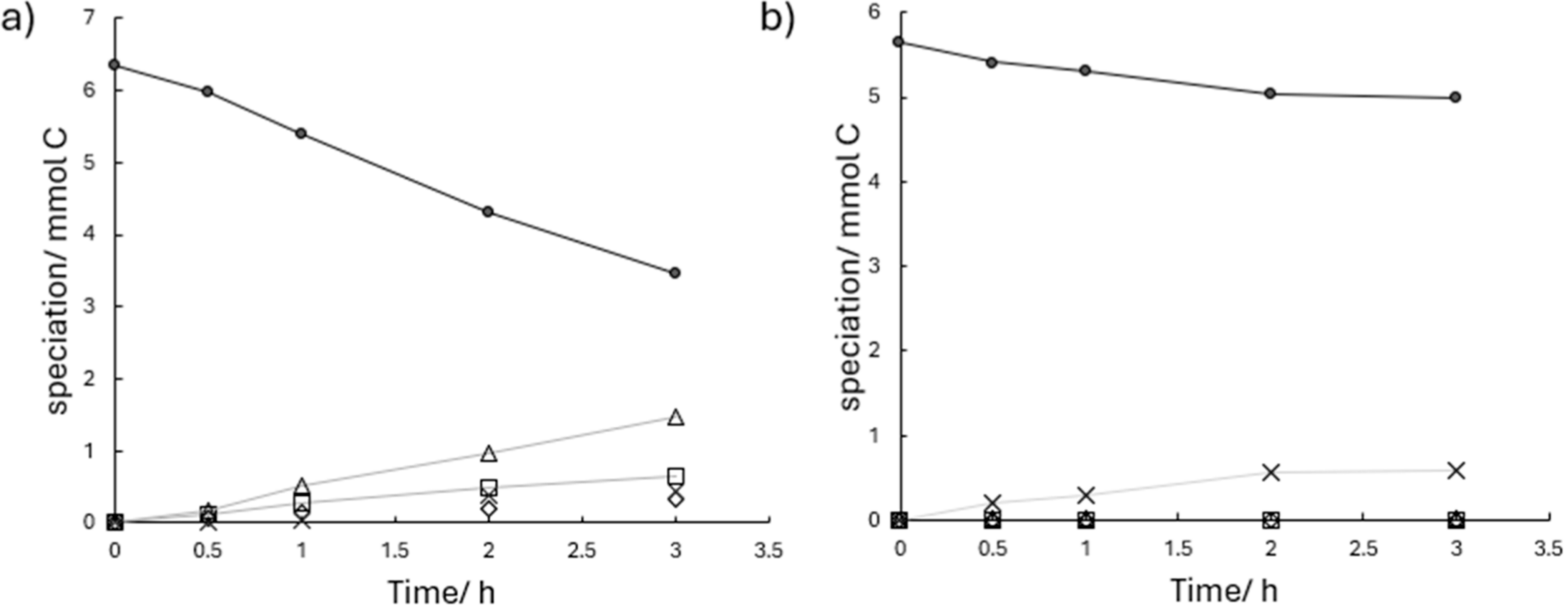
Decay of phenol and formation of byproducts during electrolysis in (a) methanolic media and (b) aqueous media. Phenol (●); non methoxylated aliphatic (□); non methoxylated aromatic (△); methoxylated (◇); carbon dioxide (X). (50 mM NaOH; *j* = 50 mA cm^–2^).

Figure 4 presents the evolution of each component within the established categories, confirming that, both in the number of species detected and in their overall abundance, electrolysis in methanolic medium is more favorable from the perspective of product formation. This is particularly relevant given that the main objective of this work is to identify pathways for waste valorization.^48,49^ A notable variety of carboxylates is generated, in agreement with other works in the literature,^50^ with oxalate, malonate, succinate, tartrate, and maleate appearing in decreasing order of importance, while other species such as fumarate are detected only at negligible concentrations. In addition, five methoxylated species are formed: 3-methoxy-1,2-benzenediol (3-methoxycatechol), 1,2-dimethoxybenzene (veratrole), 1,2,3-trimethoxybenzene, methoxybenzene (anisole), and dimethyl maleate. All these intermediates are of interest, with dimethyl maleate being the most favored product and anisole the least. With respect to the carboxylates, the application of separation and purification techniques such as electrodialysis should enable the recovery of purified carboxylate solutions, which can subsequently be processed following the electrorefinery schemes proposed in the literature.^30,31,51,52^ It is also important to note the formation of formate resulting from methanol oxidation, which would be recovered together with these carboxylates.

**Figure 4:**
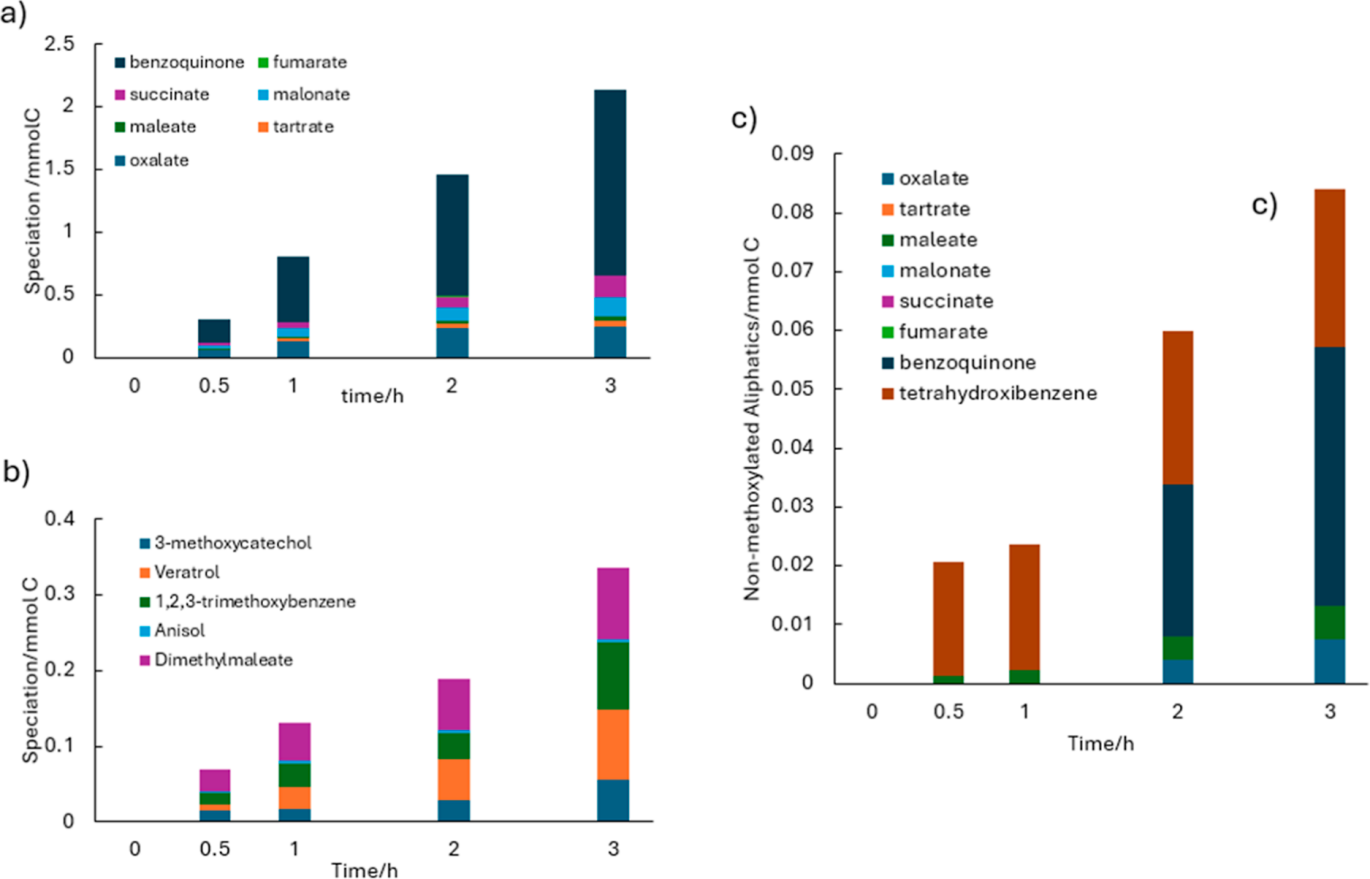
Time-dependent speciation of byproducts formed during phenol electrolysis in methanolic and aqueous media: (a) nonmethoxylated species in methanolic medium, (b) methoxylated species in methanolic medium, and (c) byproducts formed in aqueous medium (*j*: 50 mA cm^–2^, NaOH: 50 mM).

As for the second category (methoxylated species), the compounds generated are currently more challenging to purify. Nevertheless, it is important to emphasize that all detected species possess relevant applications, making future studies on their selective recovery and purification worthwhile. Dimethyl maleate serves as a monomer and chemical intermediate in the synthesis of polymers and coatings; veratrole is a bioactive compound used as an antioxidant and as a precursor to vanillin; anisole is a key intermediate in perfume production; and 1,3-dimethoxybenzene and 1,2,4-trimethoxybenzene are intermediates in the manufacture of resins, dyes, and fragrances. Another detected intermediate is benzoquinone, well-known as one of the first species formed during electrochemical phenol oxidation in aqueous media.^53–55^

Regarding oxidation in the aqueous medium, the amount of carboxylates formed, excluding formate, is negligible (0.013 mmol C) compared with the quantity generated in the methanolic medium (0.657 mmol C). This makes the recovery of these components far less attractive, as oxidation proceeds much more efficiently in water and leads predominantly to mineralization. With respect to aromatic intermediates, in addition to benzoquinone, significant amounts of tetrahydroxybenzene (a phenol molecule hydroxylated at three additional positions) were detected, further highlighting the prominent role of hydroxyl radicals generated during this process.^56,57^ However, the total amounts of these aromatic intermediates (0.071 mmol C) are also much lower than those detected in methanolic medium (1.474 mmol C), again indicating the strong influence of the electrolyte medium and the suitability of using methanol for processes aimed at recovering organic components as a consequence of deprioritizing mineralization.^22–25^ Considering all these observations, the carbon balances (in mmol of C) obtained after 3 h of treatment are summarized in Figure 5. Carbon balances were calculated by comparing the total carbon initially introduced as phenol with the carbon contained in the identified products at each electrolysis time. For this purpose, the concentration of each detected compound was converted into carbon equivalents according to the number of carbon atoms in its structure, and the sum of these contributions was expressed relative to the initial carbon content of phenol.

**Figure 5:**
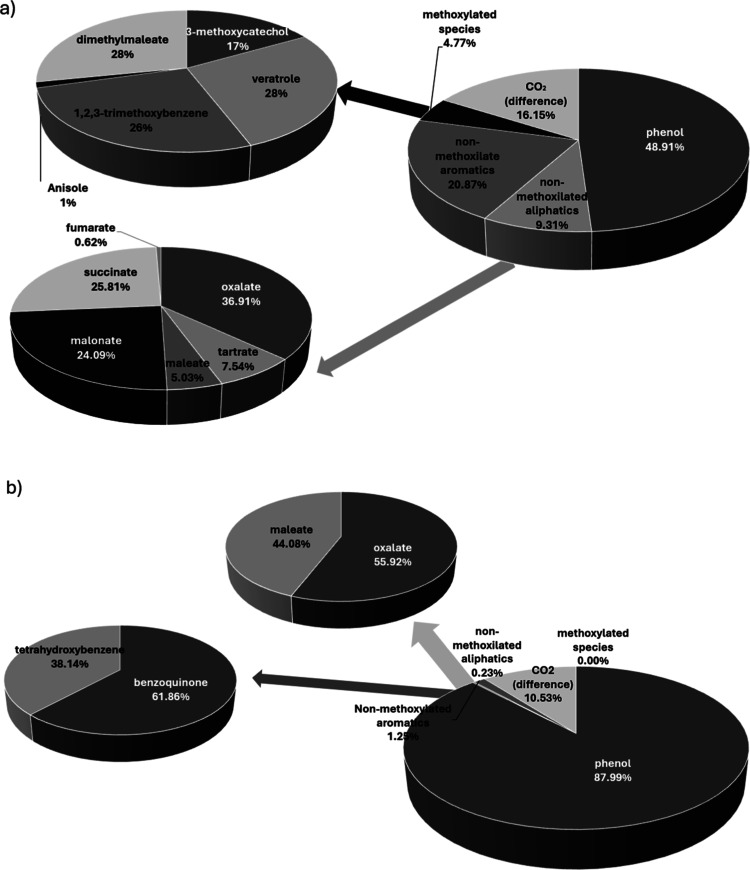
Percentage distribution of phenol and oxidation byproducts after 3 h of electrolysis in (a) methanolic medium and (b) aqueous medium (*j*: 50 mA cm^–2^, NaOH: 50 mM).

### 3.3 Phenol Degradation in a Divided Electrochemical Cell

To better distinguish the contribution of each process, electrochemical reactors compartmentalized with anion-exchange membranes (AEM) and proton-exchange membranes (PEM) were employed in a membrane electrode assembly (MEA) configuration, and the resulting performance was compared. To ensure meaningful comparability, the total electrolyte volume processed in the single-compartment reactors was proportionally divided between the two circuits of the separated cell, such that the specific electrical charge passed (eq 3), which depends solely on current intensity, electrolysis time, and treated volume, remained constant.

3 $$Q\left({Ahdm}^{-3}\right)=\frac{\int Idt}{V}$$

As shown in Figure 6, the inclusion of membranes significantly affects the process, with treatment being less effective when PEM membranes are placed between the anode and cathode, as these limit the amount of phenolate that can be oxidized at the anode.

**Figure 6:**
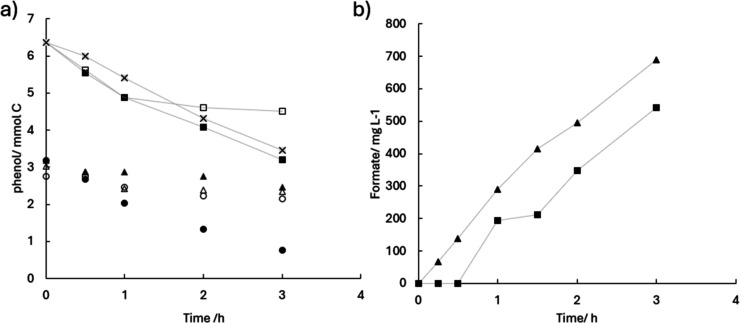
(a) Decay of phenol concentration during electrolysis in methanolic medium in a nondivided cell (×), and of the total phenol concentration in divided cells equipped with AEM (■) and PEM (□). In the divided cells, phenol decay in the anodic compartment is represented by AEM (▲) and PEM (△), while that in the cathodic compartment is represented by AEM (●) and PEM (○). (b) Evolution of formate concentration during electrolysis in divided cells equipped with AEM (▲) and PEM (■) (*j*: 50 mA cm^–2^, NaOH: 50 mM).

This behavior is not observed when AEM membranes are used, because phenol present in the cathodic compartment can migrate across the membrane and be oxidized in the anodic compartment.^58^ This explains the sharp decrease in phenol concentration measured in the cathodic compartment with AEM (and the smaller decrease observed with PEM, likely attributable to limited crossover), as well as the lower overall efficiency in phenol degradation, probably associated with mass-transport limitations.^51,59,60^ It is also important to note that no appreciable amounts of intermediates were detected in the cathodic compartment in any case, indicating that any diffusion or crossover that may occur results in concentrations below the chromatographic detection limit. Furthermore, the amount of formate generated is slightly lower in electrolysis performed with membranes, with Faradaic yields of 57.7% and 45.3% for AEM and PEM, respectively, in the production of this carboxylate.

Mineralization is substantially lower in the methanolic medium than in the aqueous medium. However, despite the higher degree of mineralization observed in water, phenol conversion remains limited under the studied conditions, reaching less than 15% after 3 h of electrolysis. In contrast, methanol promotes a more efficient degradation of organic contaminants, as widely reported in the literature. Consistently, the methanolic medium exhibited a much higher phenol conversion, exceeding 50% after 3 h.^22,25,43^

This behavior can be explained by the fact that complete mineralization requires the transfer of a much higher electrical charge: 28 electrons are needed to oxidize a single phenol molecule to CO_2_. Therefore, a substantial fraction of the applied current in the aqueous medium is consumed in driving total oxidation rather than in generating intermediate organic species. In contrast, methanol acts simultaneously as a solvent and as a reactive cosubstrate, modulating the nature of the oxidizing species and favoring partial oxidation pathways. Under these conditions, a significant portion of the applied current is directed toward the transformation of phenol into stable organic intermediates, such as carboxylates and methoxylated aromatic compounds, instead of promoting its complete oxidation to CO_2_. Thus, although phenol conversion is markedly higher in the methanolic medium, the shallower extent of oxidation accounts for the lower mineralization levels observed compared with aqueous systems.^16^

### 3.4 Comparative Overview of Byproduct Formation and Relative Distribution in Single- and Divided-Cell Systems

In the final section of this work, these efficiencies will be compared in detail, highlighting the trends described above. The final speciation results, expressed in mmol of carbon, are presented in Figure 7, where the pronounced influence of both membrane type and reaction medium becomes evident. With respect to byproduct distribution, the results show that the membrane exerts a major effect on the selectivity between methoxylated and nonmethoxylated compounds.

**Figure 7:**
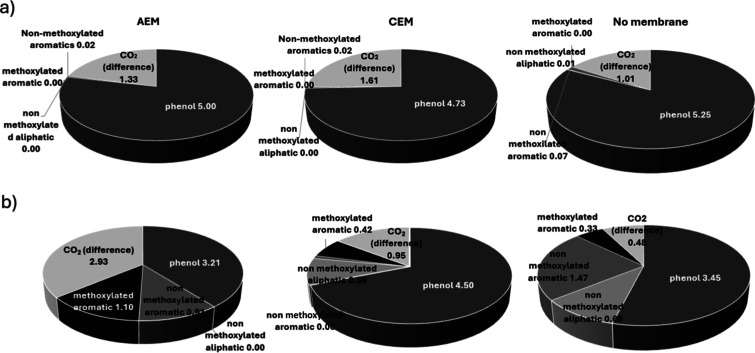
Final distribution of phenol and oxidation byproducts after 3 h of electrolysis in cells equipped with AEM, PEM, or no membrane, in (a) aqueous medium and (b) methanolic medium (*j*: 50 mA cm^–2^, NaOH: 5 mM).

In the AEM system, high concentrations of methoxylated species are observed, indicating that partial oxidation pathways dominate while mineralization proceeds in parallel. The substantial formation of methoxylated compounds can be attributed to the high availability of phenolate in the anodic compartment^58,59^ and to the presence of methanol, which promotes electrochemical methoxylation reactions and stabilizes partially oxidized aromatic intermediates. Simultaneously, the enhanced ion transport and the greater anodic efficiency in this configuration allow a significant fraction of phenol to undergo further oxidation toward CO_2_. In contrast, in the PEM system, the transport of anionic species to the anode is severely restricted, which diminishes the concentration of phenolate available at the electrode–electrolyte interface.^51,58,59^ Under these transport-limited conditions, phenol oxidation becomes governed primarily by diffusion rather than by migration, reducing the surface coverage of adsorbed aromatic species. As a result, both methoxylation reactions and deeper oxidation pathways are less favored, leading to moderate formation of methoxylated compounds as well as limited conversion to smaller organic molecules, nonmethoxylated intermediates, or CO_2._

Finally, in the absence of a membrane, a clear preference for the formation of nonmethoxylated compounds is observed, accompanied by low production of both methoxylated species and CO_2_. The lack of compartmentalization results in a less controlled electrochemical environment in which species generated at both electrodes coexist. This situation reduces the effective electrical driving force for phenolate migration toward the anode, thereby limiting its surface accumulation and, consequently, the likelihood of selective oxidation pathways. As a result, homogeneous reactions occurring throughout the bulk solution, as well as nonselective surface oxidations, are favored—leading predominantly to partial phenol transformations without efficient incorporation of methoxy groups or substantial progression toward mineralization.

Overall, these results demonstrate that the interplay between directed ion transport, reagent availability at the electrode surface, and adsorption phenomena at the anode is crucial for controlling process selectivity.^60^ The AEM configuration maximizes these effects by promoting a high local concentration of phenolate at the anodic interface,^58^ which accounts for its ability to simultaneously generate high-value products and achieve higher degrees of oxidation. In contrast, PEM operation and, even more markedly, the absence of a membrane lead to increasingly transport-limited regimes with reduced selectivity.^51,59^

### 3.5 Proposed Phenol Degradation Pathways: Formation of Carboxylates and Methoxylated Compounds

Taking all this into account, the proposed degradation pathways are shown in Figure 8 as a possible mechanism occurring within the cell. In aqueous media (Figure 8a), oxidation predominantly follows well-established pathways governed by highly oxidizing species such as hydroxyl radicals.^61,62^ Phenol is first oxidized to benzoquinone, which remains in equilibrium with hydroquinone, and then undergoes successive hydroxylation steps until the aromatic ring is ultimately cleaved. This ring-opening leads to the formation of carboxylate species such as maleate and succinate. Subsequent molecular fragmentation generates oxalate and maleate, which further degrade to oxalate; upon continued oxidation, this species ultimately yields CO_2_.

**Figure 8:**
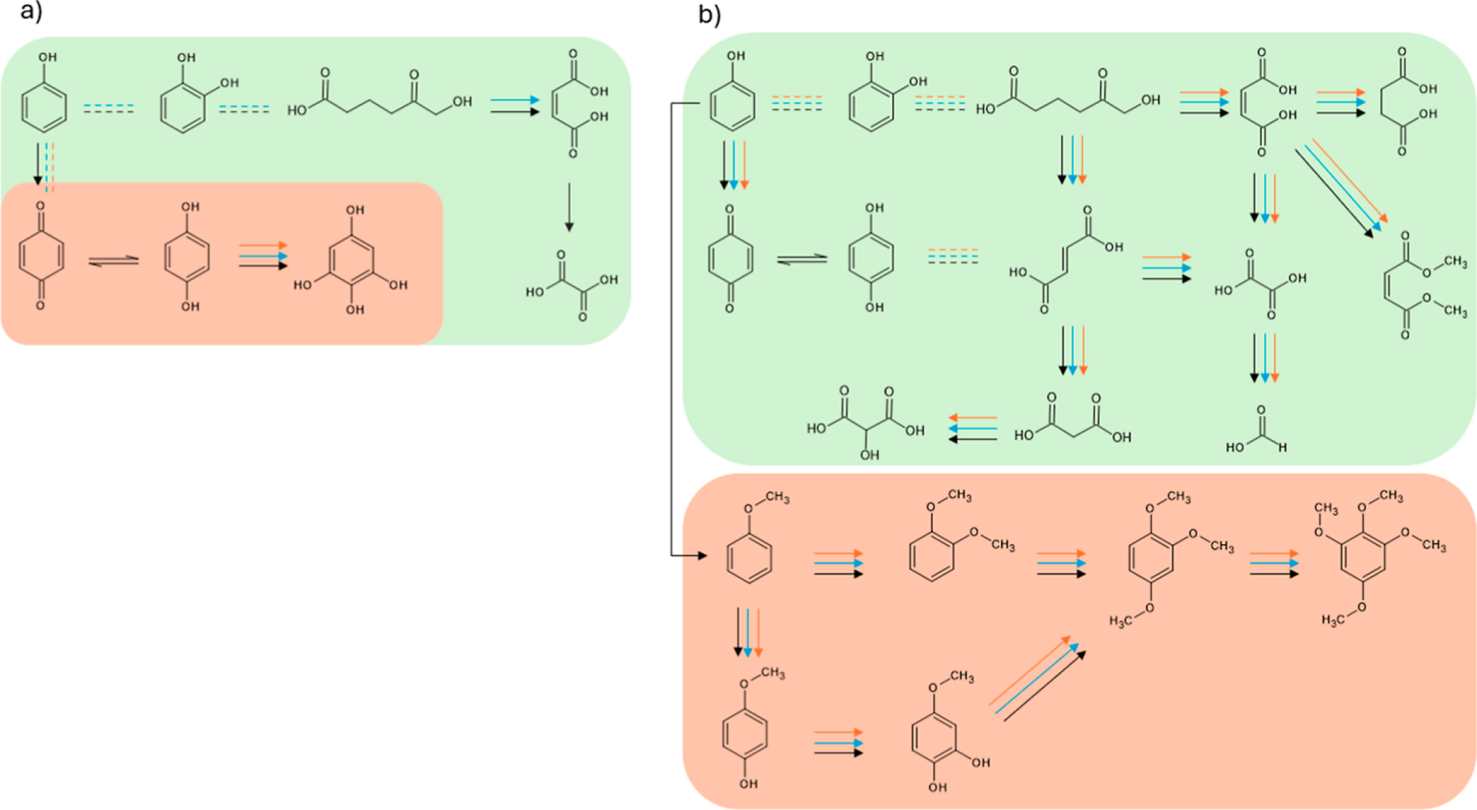
Proposed degradation pathway of phenol during electrolysis in (a) aqueous and (b) methanol media using (-) AEM, (-) PEM and (-) nonmembrane cell (green: products formed through oxidation and aromatic ring opening; light orange: products formed through hydroxylation or methoxylation of the aromatic ring).

In contrast, the methanolic medium presents a much more complex and selective reaction landscape (Figure 8b). In addition to the hydroxylation and ring-opening pathways observed in water (green area), the electrochemical oxidation of methanol generates methoxy radicals (^•^OCH_3_), which introduce additional reaction routes. These radicals can attack the aromatic ring at different positions, with the orientation and selectivity of substitution strongly influenced by the electronic effects of existing substituents, such as hydroxyl or methoxy groups already present on the ring. As a result, electrophilic substitution and radical addition reactions preferentially occur at activated ortho and para positions, leading to the formation of anisole, veratrole, and higher methoxylated derivatives, including trimethoxybenzenes (orange area).

This mechanistic framework is consistent with the detection of multiple methoxylated species and underscores the role of methanol not only as a solvent but also as a reactive participant in the oxidation process.^22–25^ Overall, these results reinforce the notion that simultaneous control of the solvent environment and ion-transport conditions is essential for shifting phenol electrooxidation away from a purely destructive approach toward an electrorefinery-based strategy focused on chemical valorization.

### 3.6 Performance Assessment Based on Faradaic and Energy Efficiencies

Another important aspect of this work concerns the Faradaic and energy efficiencies discussed throughout the manuscript, whose values are summarized in Figure 9. To evaluate these metrics, the change in the theoretical oxygen demand (ThOD) associated with each product formed was quantified and compared to the initial theoretical oxygen demand (excluding methanol and its previously discussed oxidation to formate). Using the stoichiometric relationship between ThOD and electron transfer, namely, 4 mmol e^–^ per mmol of ThOD, the corresponding Faradaic efficiencies were calculated.^63^

**Figure 9:**
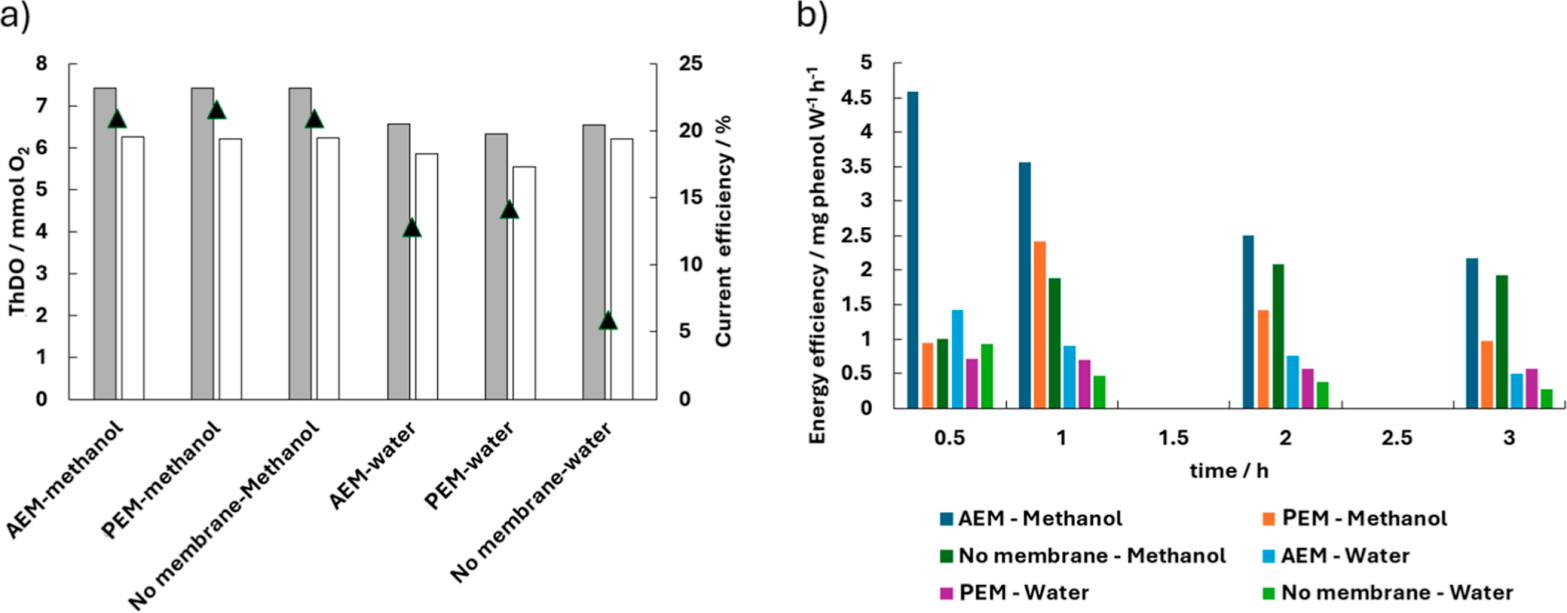
(a) Initial and final ThOD and corresponding current efficiency (▲) for oxidation products (excluding formate) under different cell configurations and reaction media. (b) Energy efficiency based on the ThOD of the oxidation products in aqueous and methanolic media.

The results are shown graphically in Figure 9a, where it can be observed that efficiencies are significantly higher in methanolic medium (slightly above 21%) compared to aqueous medium, where they are clearly below 14%, highlighting the lower performance of the process in aqueous media. Figure 9b shows that energy efficiency critically depends on both the electrolytic medium and the membrane type. In methanolic media, the AEM configuration exhibits the highest energy efficiency at the initial stages, followed by a gradual decrease over time, which can be attributed to the progressive consumption of phenol and the accumulation of reaction intermediates. Nevertheless, even after 3 h, AEM maintains the highest efficiencies, with final values of approximately 2.2 mg phenol·W h^–1^, confirming its superior overall performance in methanol compared with the PEM and no-membrane systems. This behavior indicates that AEM promotes more selective and energy-efficient oxidation pathways, associated with the electromigration of phenolate species toward the anode, where oxidation occurs with lower energy demand. In contrast, the PEM configuration exhibits the lowest efficiencies in methanol. Although the energy efficiency initially increases, it subsequently decreases, suggesting a transient regime in which phenol oxidation is temporarily favored, followed by limitations in anionic species transport and an increase in competitive reactions that reduce energy utilization. The no-membrane system shows intermediate performance, with energy efficiency gradually increasing and then stabilizing around 1.9–2.0 mg phenol W h^–1^, indicating a more stable operating regime. This behavior benefits from the absence of additional ionic resistance but lacks the selective ion transport provided by AEM^6058^.

In aqueous media, energy efficiencies are globally lower for all configurations, reflecting that phenol oxidation is dominated by hydroxyl radicals, which primarily lead to mineralization and therefore require higher energy consumption per unit of phenol removed. In addition, a significant fraction of the applied current is diverted to parasitic reactions, such as oxygen evolution, explaining the low energy efficiency values observed in water. Even so, AEM still outperforms PEM and the no-membrane system, confirming that cell compartmentalization and directed transport of charged species remain relevant in aqueous systems. The PEM and membrane-free configurations exhibit similar or lower efficiencies due to reduced selectivity and stronger competition from parasitic reactions. Overall, the results demonstrate that methanolic media combined with AEM maximize energy efficiency, whereas in aqueous systems the differences among configurations are attenuated by the radical-driven nature of the oxidation process.^25,43^

## 4 Conclusions

From this work, the main conclusions drawn are

- In methanolic electrolytes, phenol degradation exceeded 50% after 3 h of electrolysis, compared to less than 15% in aqueous systems, attributed to the different reaction pathways promoted by methanol, which favors conversion into diverse intermediate compounds rather than complete mineralization, aligning with the concept of electrorefinery for generating value-added chemicals.
- Unlike aqueous systems, where hydroxyl radical-driven oxidation tends to promote rapid mineralization, the methanolic medium provides more favorable conditions for desorption from activated carbon and facilitates methoxylation reactions. As a result, a broader spectrum of products is formed, indicating that methanol may be an interesting alternative medium for electrochemical processes focused on selective product formation, with possible future implications for valorization rather than solely pollutant destruction.
- Methanolic electrochemical oxidation generated high-value products such as dimethyl maleate, veratrole, anisole, and other methoxylated benzenes with high commercial relevance. Furthermore, carboxylates such as oxalate or maleate are also produced significantly, demonstrating the potential to transform pollutants into industrial feedstocks within electrorefinery schemes.
- Methanol oxidation to formate was a major side reaction, reaching >1 g L^–1^ with Faradaic efficiencies near to 60%, competing with phenol oxidation but offering an additional valorized product, reinforcing the multifunctional potential of methanolic electrochemical systems.
- Compartmentalized reactors revealed that anion exchange membranes (AEM) allow phenolate migration enabling oxidation and higher removal rates. In contrast, proton exchange membranes (PEM) restrict this transport, reducing degradation efficiency and limiting intermediate formation. Faradaic yields for formate were also higher with AEM (57.7%) than PEM (45.3%), confirming the critical role of membrane selection in optimizing electrochemical processes.
- The methanolic environment shifts the process away from complete mineralization and toward more selective conversion, favoring the formation of carboxylates and methoxylated aromatic compounds. In this sense, the results support the electrorefinery concept at the level of selective product formation and suggest potential future applications combining environmental remediation with valorization strategies.

## Supplementary Material



## Notes

Supporting Information details the chemicals and analytical methodologies employed to identify and quantify phenol degradation products. It describes the HPLC and LC–MS methods used to monitor phenol removal, carboxylic acid formation, and byproduct identification, including chromatographic and ionization conditions. In addition, it presents the carbon balance calculation approach, accounting for methanol-derived methoxy groups, and representative LC–MS spectra supporting compound identification and the proposed degradation pathway (PDF)

## Notes

Declaration of generative AI and AI-assisted technologies in the writing process: During the preparation of this work the authors used Co-Pilot in order to improve English and readability. After using this tool, the authors reviewed and edited the content as needed and took full responsibility for the content of the publication.
