# Supplementary material for: Beyond Treatment: Electrochemical Phenol Oxidation in Methanol under Different Cell Configurations and Its Implications for Valorization
Source: ACS Omega. 2026 Apr 30;11(18):27481–92. doi: 10.1021/acsomega.6c01902 (PMC13177212; doi:10.1021/acsomega.6c01902)
Supplement: Supplementary file 1 [file ao6c01902_si_001.pdf]

Supporting information

Beyond Treatment: Electrochemical Phenol Oxidation in Methanol under Different Cell Configurations and Its Implications for Valorization

William Santacruz[1,2], Cristina Navas-Higuero[2], Cristina Saez[2], Artur de Jesus Motheo[1] and Manuel Andrés Rodrigo[2,*]

[1] São Carlos Institute of Chemistry, University of São Paulo, São Carlos - SP, 13566-590, Brazil

[2] Department of Chemical Engineering, Universidad de Castilla-La Mancha, Ciudad Real 13071, Spain

Corresponding Author:

*E-mail address: manuel.rodrigo@uclm.es

Experimental Procedures

2.1. Chemicals

Phenol (≥99.5%, Sigma-Aldrich), methanol (99.9%, PanReac), and sodium hydroxide (Scharlau) were used for degradation experiments. Acetonitrile (≥99.9%, Honeywell HPLC grade) and sulfuric acid (97%, Scharlau) were used for High-Performance Liquid Chromatography (HPLC) analysis. For Liquid Chromatography-Mass Spectrometry (LC-MS), methanol (LC-MS grade, Fisher Chemical), ammonium acetate (Merck), and formic acid were employed.

The following reagents were used as standards for identification: anisole (99%), dimethyl maleate (96%), maleic acid (99%), and oxalic acid (99%) from Thermo Scientific; fumaric acid (99%) and 1,4-benzoquinone from PanReac; succinic acid (99%) and malonic acid (99%) from Sigma-Aldrich; 1,2-dimethoxybenzene (veratrole) and 1,2,4-trimethoxybenzene from TCI; 4-methoxybenzene-1,2-diol (Fluorochem); tartronic acid (97%, Fluka Analytical); and formic acid (99%, Normapur). All chemicals were used as received without further purification. Deionized water (Millipore Milli-Q, resistivity 18.2 MΩ cm^{-1}) was used throughout the study.

2.3. Characterization methodology

The phenol removal and carboxylic acids generation were evaluated by high-performance liquid chromatography (HPLC) with an Agilent 1260 Infinity III system, which was connected to a UV detector at 272 nm or 210 nm, respectively. To assess the phenol removal, the chromatographic separation was achieved using a Kinetex EVO (150 x 4.6 mm x 5µm) reversed-phase C18 column. The mobile phase consisted of a 70:30 v/v mixture of 0.1% formic acid solution and acetonitrile and it was flowed at as a flow rate of 1.0 mL min^{-1} at 25 °C. For the quantification of carboxylic acid generated, it was used an Agilent Hiplex-H reversed-phase column (300 × 7.7 mm). The mobile phase was 5 mM sulfuric acid, flowed at 0.6 mL min^{-1} at a temperature setting of 50 °C.

For LC-MS an Agilent 1260 Infinity coupled with a 6230 TOF was used, equipped with an Agilent Infinity Lab Poroshell 120 EC-C18 column (3.0 x 50 mm x 2.7 μm) and with an electrospray ionization source. The ionization source parameters were set as follows: spray voltage, 3500 V; curtain gas temperature, 325°C; curtain gas flow, 8.0 L min^{-1}; nebulizer gas flow, 35 psi; drying gas, 3 L min^{-1}. The mobile phase consisted of a mixture of methanol (A) and a solution of 5 mM ammonium acetate with 0.1% v/v formic acid (B). The mobile phase started as 30% A and 70% B, followed by a linear gradient to 90% A in 3 min, and kept isocratic for 2.5 min, and then back to 30% A for 0.5 min. A flow rate of 0.2 mL min^{-1} was used, and the injection volume was 2 μL. Temperature was fixed at 35°C

Results and Discussion

- *Carbon balance calculation:*

The carbon balance was expressed as the percentage of phenol-derived carbon distributed among the detected products relative to the initial carbon introduced as phenol. For this purpose, the amount of carbon associated with each identified product was calculated from its concentration and molecular formula. In methanolic medium, special care was taken with methoxylated aromatic products, since part of their carbon content does not originate from phenol but from methoxy groups incorporated from methanol. Therefore, for these compounds, the carbon corresponding to the methoxy substituents was corrected separately and not attributed to phenol-derived carbon (Eq. S1-S3).

Accordingly, the phenol-derived carbon remaining in aromatic products was obtained by subtracting the carbon associated with aliphatic by-products, aromatic by-products, and methoxylated aromatic by-products from the initial phenol carbon, while adding back the carbon introduced through methoxy groups derived from methanol. The non-identified fraction of phenol-derived carbon was assigned by difference and expressed as CO_2 equivalent. All carbon balance values were finally expressed as percentages relative to the initial phenol-derived carbon (Eq. S2).

$$C_{\text{final}} = C_{\text{phenol,0}} - C_{\text{aliphatics}} - C_{\text{aromatics}} - C_{\text{methoxylated}} + C_{\text{methoxy added}} \quad \text{(S1)}$$

where: C_{final} = phenol-derived carbon remaining during electrolysis; $C_{\text{phenol,0}}$ = initial carbon introduced

as phenol; C $_{aliphatic}$ = carbon quantified in detected aliphatic by-products; C $_{aromatics}$ = carbon quantified in detected non-methoxylated aromatic by-products; C $_{methoxylated}$ = total carbon quantified in detected methoxylated aromatic by-products; C $_{methoxy\ added}$ = carbon corresponding only to the methoxy groups incorporated from methanol. All values expressed in mmol of C.

And then, for the CO_2 equivalent:

$$C_{CO_2,eq} = C_{phenol,0} - C_{final} \quad (S2)$$

When expressed as a percentage for each by-product (C_i):

$$C_{fraction}\ (\%) = \frac{C_i}{C_{phenol,0}} \times 100 \quad (S3)$$

- *Spectra obtained by Liquid Chromatography coupling with mass spectrometry (LC-MS)*

Representative LC–MS spectra of the by-products identified during phenol degradation in methanolic medium are shown below. One spectrum for each detected compound is provided to support compound identification and the proposed degradation mechanism.

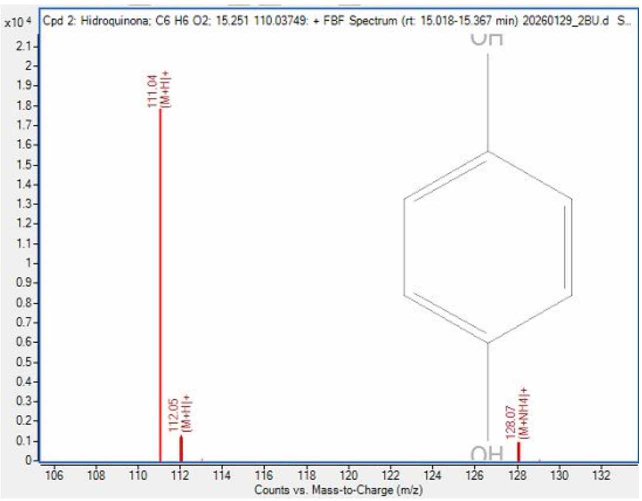

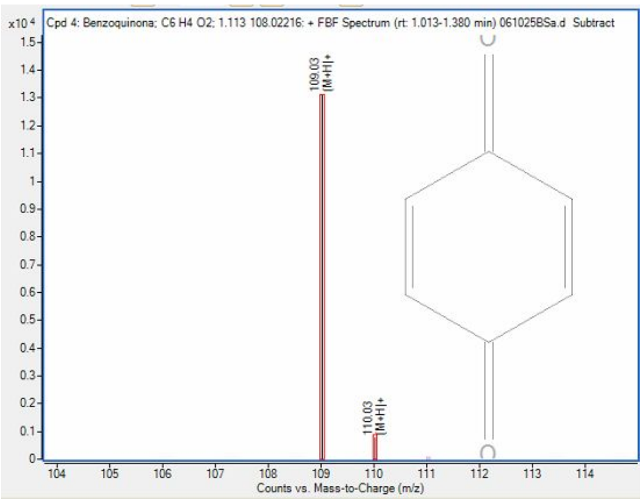

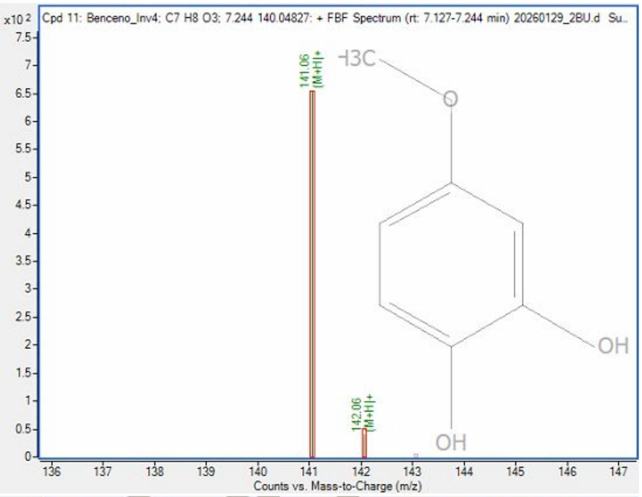
Cpd 11: Benceno_Inv4; C7 H8 O3; 7.244 140.04827: + FBF Spectrum (rt: 7.127-7.244 min) 20260129_2BU.d Su...
141.06 [M+H]+
142.06 [M+H]+
Counts vs. Mass-to-Charge (m/z)

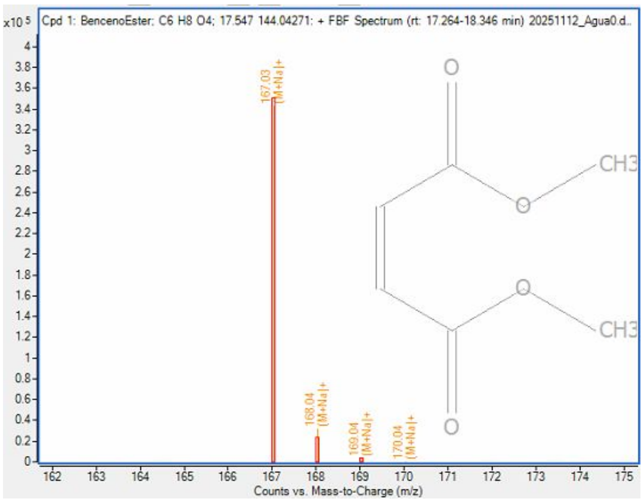
Cpd 1: BencenoEster; C6 H8 O4; 17.547 144.04271: + FBF Spectrum (rt: 17.264-18.346 min) 20251112_Agua0.d...
167.03 [M+Na]+
168.04 [M+Na]+
169.04 [M+Na]+
170.04 [M+Na]+
Counts vs. Mass-to-Charge (m/z)

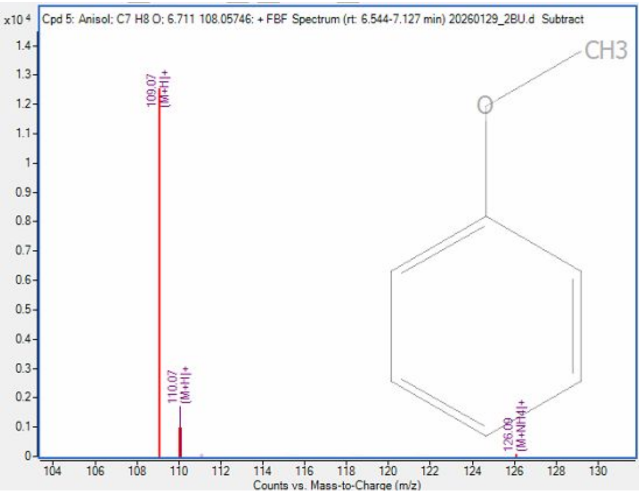
Cpd 5: Anisol; C7 H8 O; 6.711 108.05746: + FBF Spectrum (rt: 6.544-7.127 min) 20260129_2BU.d Subtract
109.07 [M+H]+
110.07 [M+H]+
126.09 [M+NH4]+
Counts vs. Mass-to-Charge (m/z)

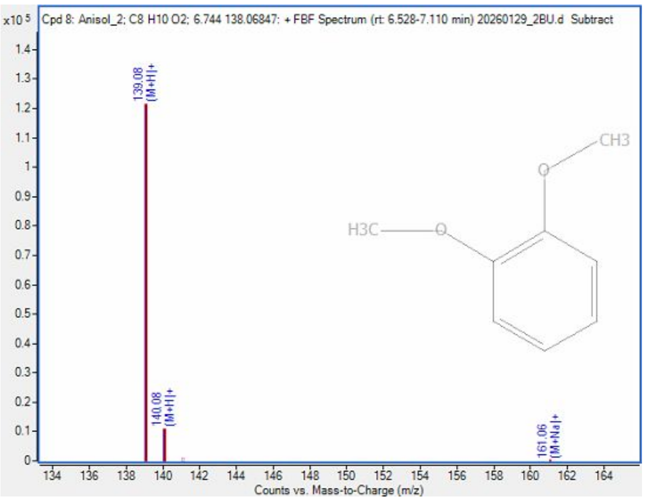
Cpd 8: Anisol_2; C8 H10 O2; 6.744 138.06847: + FBF Spectrum (rt: 6.528-7.110 min) 20260129_2BU.d Subtract
139.08 [M+H]+
140.08 [M+H]+
161.06 [M+Na]+
Counts vs. Mass-to-Charge (m/z)

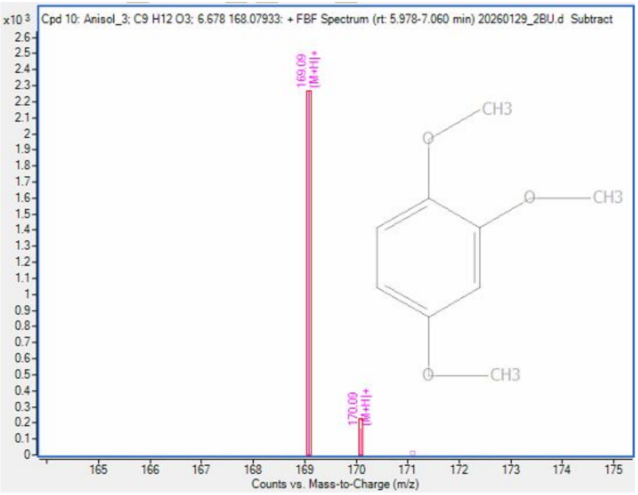
Cpd 10: Anisol_3; C9 H12 O3; 6.678 168.07933: + FBF Spectrum (rt: 5.978-7.060 min) 20260129_2BU.d Subtract
169.09 [M+H]+
170.09 [M+H]+
Counts vs. Mass-to-Charge (m/z)

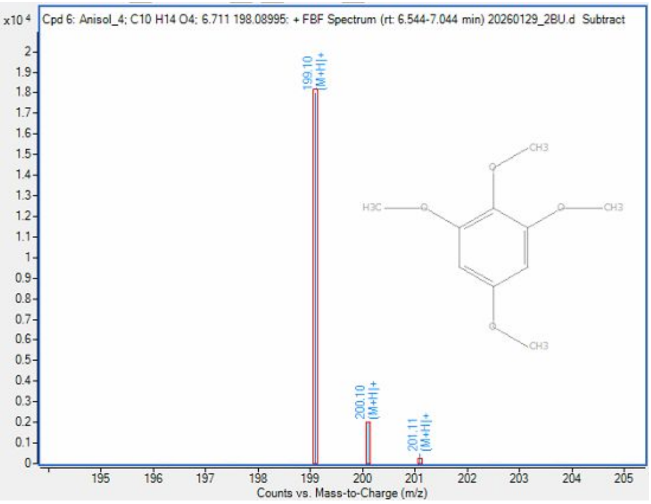
Cpd 6: Anisol_4; C10 H14 O4; 6.711 198.08995: + FBF Spectrum (rt: 6.544-7.044 min) 20260129_2BU.d Subtract
199.10 [M+H]+
200.10 [M+H]+
201.11 [M+H]+
Counts vs. Mass-to-Charge (m/z)
